# Troublesome Crystal Structures: Prevention, Detection, and Resolution

**DOI:** 10.6028/jres.101.034

**Published:** 1996

**Authors:** Richard L. Harlow

**Affiliations:** Central Research and Development E. I. Dupont de Nemours & Co., Inc. Wilmington, DE 19880-0228

**Keywords:** fuzzy structures, incorrect structures, *R* value, single-crystal structures, thermal parameters

## Abstract

A large number of incorrect crystal structures is being published today. These structures are proving to be a particular problem to those of us who are interested in comparing structural moieties found in the databases in order to develop structure-property relationships. Problems can reside in the input data, e.g., wrong unit cell or low quality intensity data, or in the structural model, e.g., wrong space group or atom types. Many of the common mistakes are, however, relatively easy to detect and thus should be preventable; at the very least, suspicious structures can be flagged, if not by the authors then by the referees and, ultimately, the crystallographic databases. This article describes some of the more common mistakes and their effects on the resulting structures, lists a series of tests that can be used to detect incorrect structures, and makes a strong plea for the publication of higher quality structures.

## 1. Introduction

The determination of crystal/molecular structures by x-ray single-crystal diffraction methods has been blessed with three characteristics that render it absolutely unique as an analytical technique. First, the technique is very robust: structures can be extracted from diffraction data even when they suffer from severe statistical and systematic errors. Second, there are numerous checkpoints along the path of a structure determination which can be used to guide one to the correct structure or, at least, guide one away from an incorrect structural model. Finally, if done correctly, the result is a very cost-effective “picture” of the atomic arrangement with a large amount of information content: bond distances, coordination geometries, thermal motions, intermolecular interactions, etc.

With the automated data collection procedures, low-temperature capabilities and computer analysis packages available today, the conversion of diffraction spots into a crystal structure ought to be perfectly straightforward for the average molecular crystal and only somewhat more difficult for inorganic structures where problems with absorption, twinning, pseudosymmetry etc., are more common. As it turns out, however, a fair number of “wrong” structures are being published each year which indicates that the authors, the reviewers, and the editors have all failed to recognize one or more symptoms of incorrect structures. My definition of an “incorrect” or “wrong” structure, by the way, is simply that one or more of the critical crystallographic outputs, namely the unit cell, the space group symmetry, the types of atoms and their positional and thermal parameters, have been improperly determined and reported. No one knows how many incorrect structures have been placed in the crystallographic databases but, in spite of repeated reviews on the subject [1,2,3], the number is obviously continuing to grow: each review contains far more examples than the previous one. The purpose of this chapter is to examine even more examples of troublesome structures in order to understand better how incorrect structures can be detected and hopefully resolved before they get into print and into the databases.

## 2. Structure Classifications

### 2.1 Quality Structures

It is useful to point out that crystal structures, in my opinion, fall into one of four classifications. First, there are *quality* structures. These are structures in which all of the atoms, including the hydrogen atoms if present, have been refined to yield reasonable positional and thermal parameters. Hydrogen atoms in organic and organometallic structures are true indicators of the quality of both the data and the structural model. I maintain that if you can refine the hydrogen atoms (isotropic thermal parameters, *B*, that are less than 6.0 Å^2^ and all of the C–H bonds are between 0.85 Å and 1.05 Å), the data must be of high quality and the model must be correct. Oxygen atoms in an inorganic oxide can serve the same purpose but the M–O distances can be more variable and the oxygen atoms, in this case, must be refined with anisotropic thermal parameters. Of course, all of the anisotropic thermal parameters should be reasonable, both in magnitude (for organic and organometallic structures, no Beq greater than 5 A^2^; for inorganic structures, no Beq greater than 2.5 A^2^) and in shape (cigar- and disk-shaped ellipsoids generally eliminate a structure from the quality classification; adjacent atoms vibrating in orthogonal directions is also an unacceptable feature). Chemically-equivalent bonds, particularly C–C bonds, should have equivalent bond distances. Finally, quality structures are, of course, characterized by good *R* (*R*=Σ||*F*_0_|–|*F*_c_||/Σ|*F*_0_|) values.

### 2.2 Fuzzy Structures

The second category, which includes most of the structures being reported today, contains the *fuzzy* structures. These are firstly and primarily characterized by good *R* values. This group contains most of the organic and organometallic structures which have been solved and refined with “room-temperature” data. Hydrogen atoms, if included at all, refine to poor positions with widely variable, generally high, thermal parameters, or are “idealized.” These structures may contain atoms with strange-looking anisotropic thermal parameters, atoms which have been constrained or restrained in some fashion, and/or “disordered” atoms. They also may have considerable variation in their chemically-equivalent bond distances and may even have bond distances which are unreasonable when compared to those in related structures.

### 2.3 Incorrect Structures

The third category consists of the *incorrect* structures. These, unfortunately, have all of the same characteristics of the *fuzzy* group, including low *R* values, and so the two are often hard to distinguish at first glance. Generally, however, their problems are more severe: improbable coordination geometries, bizarre bond distances and angles, impossible intra- and intermolecular contacts, and nonpositive-definite anisotropic thermal parameters.

### 2.4 Junk Structures

The last category includes all the structures with high *R* values. A low *R* value, characteristic of correct as well as incorrect structures, obviously has very little meaning, but a high *R* value sends a clear message that something is very wrong. The adjective “high” is both structure dependent, i.e., what types of atoms are present, and procedure dependent, i.e., were all the data or only the “observed” data used in the refinements? Nonetheless, structures with *R* values well above 0.15 (based on “observed” reflections!) continue to be published. I have decided to exclude structures in this category from further consideration because there are no useful lessons to be learned from them (unless, of course, you’re interested in publishing such a structure and want to know which journals still accept them).

## 3. Examples of Troublesome Structures

I have tried to select a wide variety of examples which emphasize particular crystallographic problems. The unique symptoms and the ultimate disposal of each case will be described briefly. Most of these examples were not included in the earlier reviews [1,2,3]: in Sec. 4, I will attempt to summarize the lessons that can be learned from all of these sources.

### 3.1 Incorrect Laue Group/Space Group Symmetry

**Fig. 1:**
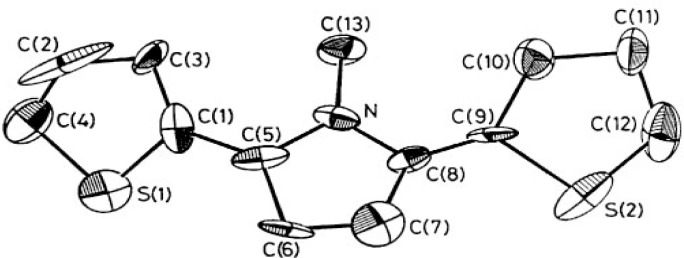
Thermal ellipsoid drawing of 2,5-*bis*(2-thienyl)pyrrole.

Probably the most common error in crystal structures relates to incorrect symmetry, particularly cases where the assigned symmetry is too low. Refinements are often done in the wrong space group and sometimes in the wrong Laue group. Marsh [4a] and Baur [4b] have published extensively on this type of mistake and by now there is a clear picture of the kind of symptoms that these structures exhibit. A particularly good example [5] is shown in Fig. 1.

This structure was refined in noncentrosymmetric space group Pca2_1_ when it should have been refined in centrosymmetric Pcam (Pbcm). Not only are the thermal ellipsoids nonsensical, but the chemically-equivalent bond distances such as S(1)–C(1) and S(2–C(9) differ by 0.10 Å. These symptoms are caused by the high correlations between positional and thermal parameters of atoms in the two halves of the molecule which are actually related by a mirror plane in Pcam. Although a re-refinement of the structure has yet to be carried out in Pcam, the mirror planes which are perpendicular to the *c*-axis can be seen quite clearly in a packing diagram.

Cases where the assigned symmetry is too high appear to be rather rare. This is probably true because the *R* values tend to “hang” at unacceptable levels which makes these structures difficult to publish. Lowering the symmetry, generally dropping from a centrosymmetric to a non-centrosymmetric space group, often resolves the problem with the *R* values. If left in the higher symmetry space group, the model is forced to fit the “average” of the two asymmetric units which are only approximately related by the imposed crystallographic symmetry. Experience in our laboratory and elsewhere [6] suggests that such a structure will either contain atoms with enlarged anisotropic thermal parameters or will appear disordered.

### 3.2 Incorrect Atom Types

**Fig. 2:**
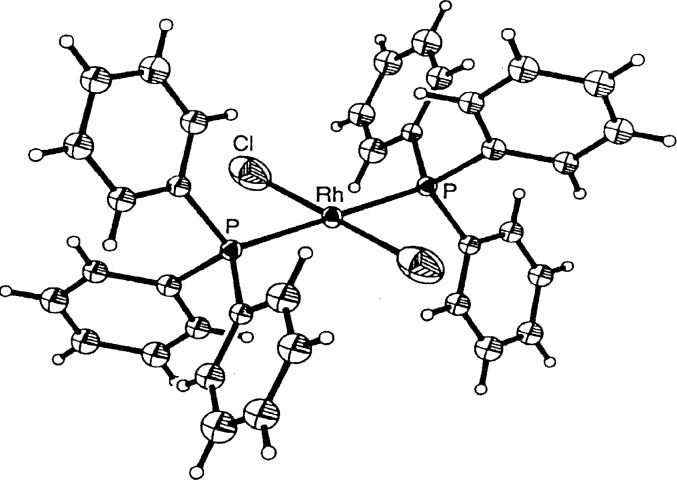
Thermal ellipsoid drawing of *trans-bis*-(triphenylphosphine)-rhodiumdichloride.

The second most common error is the incorrect identification of atom types. The next structure was reported to be the first monomeric rhodium(II) complex containing two phosphine and two chloride ligands [7]. If true, one would have to explain why the Rh–Cl bonds, 2.428 Å, are much longer than expected and why the chloride ligands have large thermal motions parallel to the Rh–Cl bond as shown in Fig. 2.

**Fig. 3:**
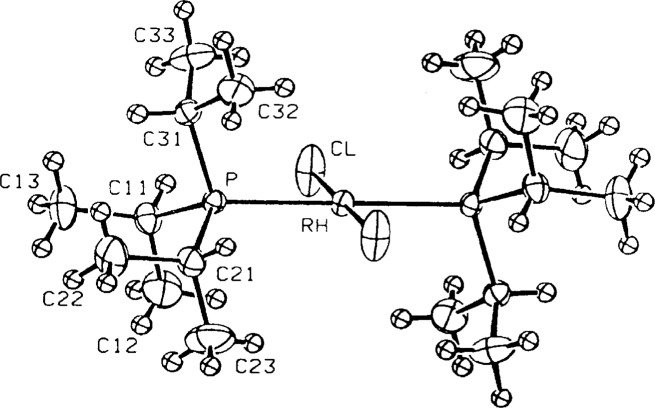
*Trans-bis*(tri-isopropylphosphine)rhodiumdichloride.

In addition, rhodium(II) should be paramagnetic and yet the ^1^H NMR spectrum is quite “normal,” indicative of a diamagnetic compound. Both of these anomalies can be explained if the compound is actually a rhodium(I) complex with one Cl and one CO (or N_2_) ligand. As a disordered structure (the Rh sits on a crystallographic inversion center), the “thermal” ellipsoid associated with the Cl atom has been forced to fit the three half-occupied C, O, and Cl atoms, necessarily elongating it in the direction of the bond. In addition, the center of the electron density has shifted away from the rhodium atom, lengthening the apparent Rh–Cl bond. This structure was recently re-examined in more detail [8]; a model based on a disordered Cl and CO refined very well. A true dichloride has also been published and is pictured in Fig. 3 [9]. It shows both a normal Rh–Cl bond of 2.298 Å and a thermal ellipsoid elongated perpendicular to the Rh–Cl axis.

**Fig. 4:**
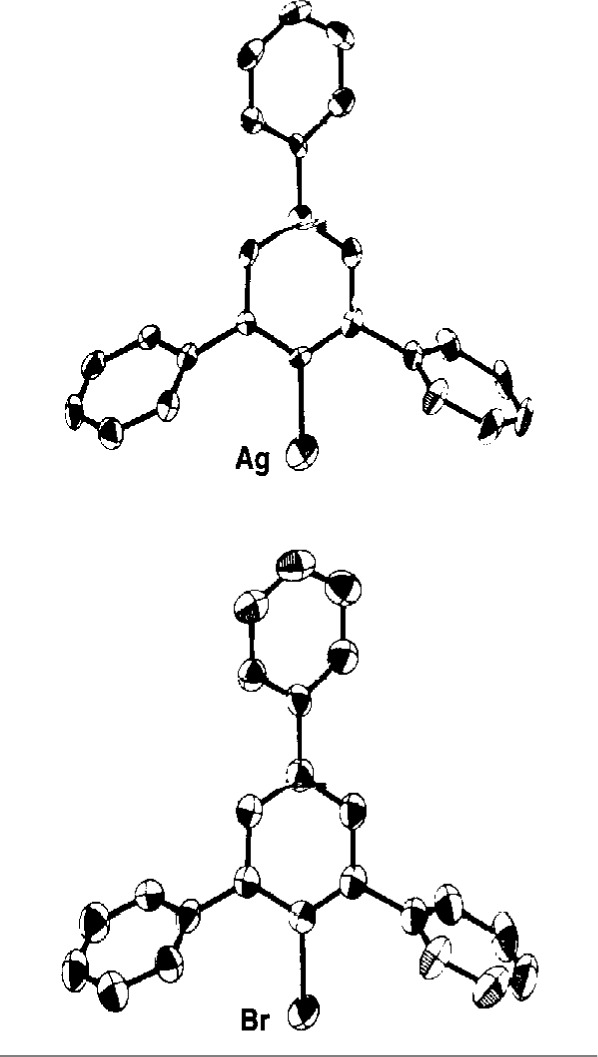
Thermal ellipsoid drawings of 2,4,6-triphenylbenzene bonded to a single atom of Br in the correct structure, Ag in the incorrect structure.

In the case just described, the thermal ellipsoid had an usual shape but not an unusual size. This is basically because the CO group has roughly the same number of electrons as the chlorine. If the identity of an atom, especially a “heavy” atom, is mistaken, the thermal ellipsoid will be proportionately larger or smaller, depending on the difference in the number of electrons between the real atom and the atom used in the model. Such was the case in two compounds with the general formula Ph_3_C_6_H_2_M, where *M* = Cu and Ag. These compounds, if correct, would have been unique because the metal atoms have a coordination number of 1! The original paper [10] was published with only a packing diagram with the molecules represented by balls and sticks. Lacking any visualization of the thermal ellipsoids, there was little indication that the atom types were incorrect except for the fact that the Cu–C and Ag–C bond lengths were virtually identical, something not expected for a first- and second-row transition metal. These structures were ultimately reinvestigated [11] and found to be bromides. Figure 4 shows both the incorrect structure refined as a Ag complex and the correct structure where the atom is refined as a Br. The thermal ellipsoid of the Ag atom, in spite of being the heaviest atom in the structure, is much larger than those of the carbon atoms.

Perhaps more insidious are the cases where one atom type partially substitutes for another. Crystallization of compounds with the general formula MoOCl_2_(PR_3_)_3_ led to both blue and green crystals. Crystal structures for a number of compounds with different *R* substituents showed the blue form had the expected structure with a short M = O distance while the green form had a much longer M–O bond, more indicative of a single bond rather than a double bond. The term “bond-stretch isomers” was coined for these complexes in which the orbitals of the Mo atom could presumably rehybridize to accommodate either a single or double bond to the oxygen atom. All of this was eventually shown to be incorrect [12]: the oxygen site in the green crystals was partially occupied by a chlorine atom. Since chlorine is a stronger scatterer than oxygen, and since a Mo–Cl bond is much longer than a Mo = O bond, it only takes a small contamination of the site to significantly lengthen the bond. It is interesting that the thermal ellipsoids of the the oxygen atoms in the green crystals do not show any significant elongation in these cases. In other examples of possible bond-stretch isomerism, in particular the NbOCl_3_(PMe_3_)_3_ series, the thermal ellipsoids did give some warning that the oxygen site was contaminated with chlorine [13].

### 3.3 Incorrect Unit Cells/Bravais Lattices

Automated indexing software on today’s diffractometers makes the job of determining a unit cell and its Bravais lattice trivial. Unfortunately, the unit cell which comes out is only as good as the reflections which are put into it. If the reflections are not representative of the true cell, wrong unit cells will result. This is of particular concern when a superlattice is present since only the strong reflections are generally used in the indexing routine. It is equally unfortunate that the autoindexing routine is often not followed up with a series of oscillation photographs to look for weak superlattice reflections or reflections which might alter the Bravais lattice. Missing a superlattice or mistakenly assuming a centered lattice results in an “average” structure in which many of the atoms may have unusually large thermal parameters and/or appear disordered.

**Fig. 5:**
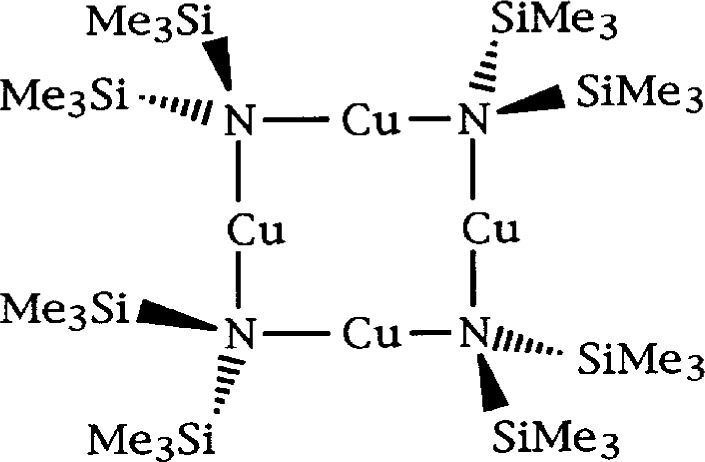
Chemical structure of a [CuN(SiMe_3_)_2_] tetramer.

Such was the case in the structure of the usual tetramer shown diagrammatically in Fig. 5. Based on “room-temperature” data, the structure was solved and refined in space group I2/m [14].

**Fig. 6:**
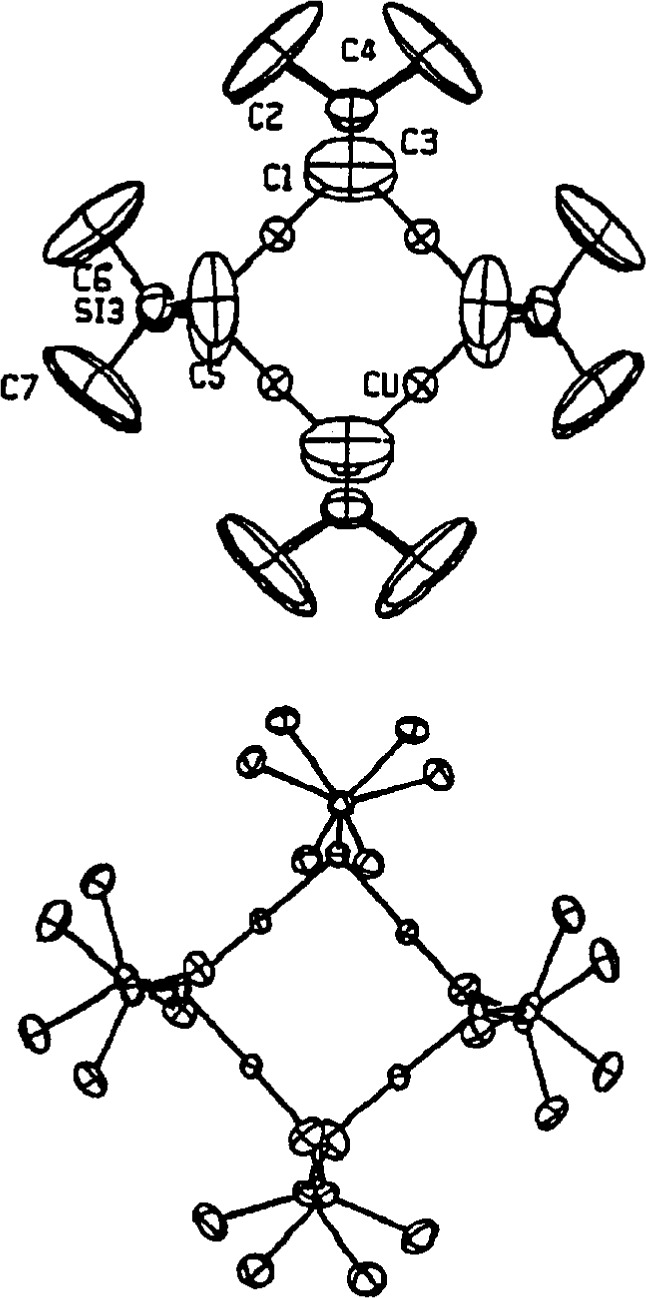
Thermal ellipsoid drawing of the [CuN(SiMe_3_)_2_] tetramer as refined in space group I2/m (top). Same structure refined in P2/n (bottom).

Although the thermal ellipsoids have not been shown, the *U*_eq_ for many of the methyl carbons are very large. A great deal of effort was expended by the authors to improve the structure by using DIFABS, applying extinction corrections, and attempting refinements in I2 and Im. None of these worked. The real problem with the structure is that the cell is not body-centered but primitive, P2/n [15]. The weak reflections, which are clearly observable even at room temperature, had been missed by the original authors. A comparison of the ORTEP drawings for the incorrect and correct structures is given in Fig. 6. In the correct structure, the methyl carbons have the expected staggered conformation; in the incorrect structure, their positions are averaged to give an eclipse conformation, the large ellipsoids being required to account for the smeared electron density (actually, this structure probably would have refined better if a disordered model had been used for the methyl carbon atoms).

**Fig. 7:**
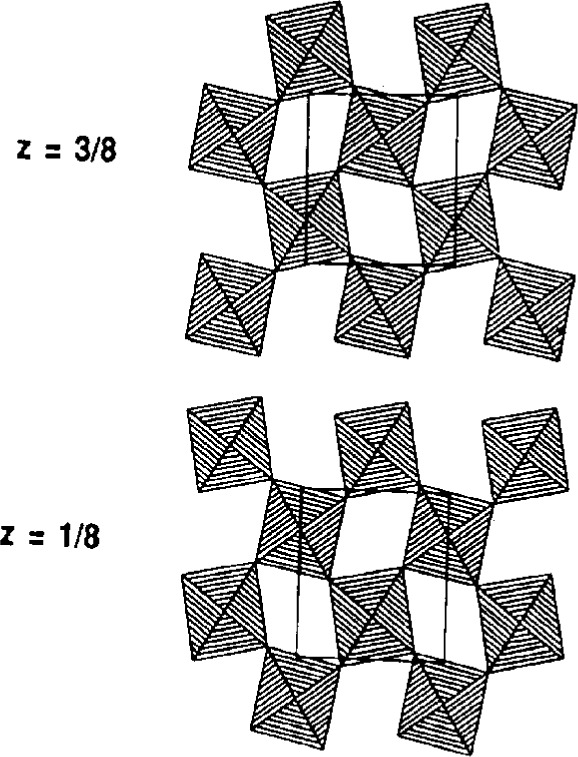
Two layers of the Sr_2_IrO_4_ structure showing the rotation of the IrO_6_ octahedra. Only the in-plane oxygen atoms contribute intensity to the superlattice reflections.

As a second example, we have investigated the structure of Sr_2_IrO_4_ which, on first appearance, crystallizes in a small tetragonal cell: *a* = 3.89 Å, *c* = 12.90 Å [16] with space group I4/mmm. Refinement of the structure showed that it consisted of sheets of IrO_6_ octahedra, corner-shared with Ir–O–Ir angles of exactly 1808. Two problems arose, however: the anisotropic thermal ellipsoid of the unique in-plane oxygen atom was severely elongated perpendicular to the Ir–Ir vector and the compound showed ferromagnetic behavior which is not possible if the compound is truly I4/mmm. Careful studies using single-crystal photographic and synchrotron powder-diffraction techniques eventually revealed seven superlattice reflections: the cell, in fact, had a volume four times that of the original cell. An investigation of a single-crystal on an in-house, sealed-tube diffractometer showed that only one of these reflections had a measured intensity above the three sigma level under “normal” operating conditions. The structure, as shown in Fig. 7, was eventually refined using neutron powder diffraction data (where the superlattice reflections were more easily seen) and yielded a structure in which the IrO_6_ octahedral layers were rotated by ±11°, making the Ir–O–Ir angle 157.9° [17].

### 3.4 Wrong Site Symmetry

**Fig. 8:**
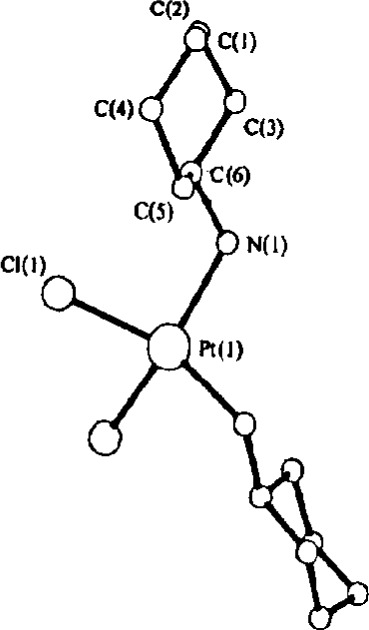
Ball-and-stick model of the presumably *cis-bis*(cyclohexylamine)-platinum dichloride when the complex was placed on a crystallographic 2-fold axis.

Many space groups offer the possibility of placing an atom on more than one type of site symmetry. On discovering that *cis*-(C_6_H_13_N)_2_PtCl_2_ crystallizes in space group Pbcm with *Z* = 4, it was quite natural to assume that the Pt atom must sit on a 2-fold axis rather than on an inversion center. The structure of this complex was subsequently solved, refined (*R* = 0.054) and published [18]. A thermal-ellipsoid drawing was not provided but the structure, as shown in Fig. 8, is quite unusual in the sense that the Pt atom adopts a flattened tetrahedral coordination rather than a square-planar geometry found for virtually all other 4-coordinate Pt(II) complexes.

**Fig. 9:**
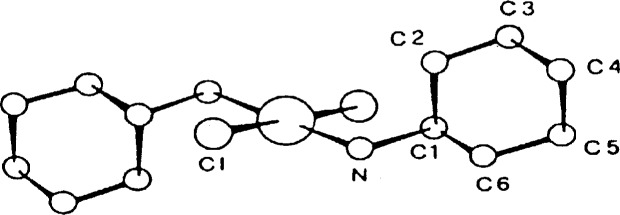
Structure of *trans-bis*(cyclohexylamine)platinumdichloride when the complex was placed on a crystallographic inversion center. The size of the atoms is arbitrary; the spheres are not thermal ellipsoids.

A year later, the structure of the trans-complex was reported [19] with no reference to the earlier work on the *cis*-complex. Its structure (*R* = 0.041) is shown in Fig. 9 and appears quite normal, at least in terms of the Pt coordination.

Several years later, the two structures were compared [20] and it was noted that they had identical lattice parameters and space groups. Clearly, the *cis*-complex was incorrect: apparently, the compound underwent an isomerization to the trans form during the recrystallization process.

### 3.5 Hydrogen Atoms

It is often claimed that hydrogen atoms are sometimes difficult to locate and refine. If authors insist on relying on “room-temperature” data, I might agree. With “low-temperature” data, however, there is very little excuse unless absorption or disorder effects cannot be adequately dealt with. Hydrogen atoms are not only important from a structural point of view (omitting them causes some shift in the positions of the atoms that they are bonded to) but also from a chemical viewpoint in terms of establishing the exact chemical formula: hydrogen bonded dimers, hydride formation, etc. One example of the latter is the structure of Cp*Co(H)_3_CoCp* which recently made headlines when it was reported as Cp*Co=CoCp*, the first true example of a metal-metal multiple bond in which there were no bridging atoms [21]. The structure was met with some skepticism and, almost immediately, a reinvestigation showed that there were three bridging hydrides which had been missed in the original study [22].

### 3.6 Polar Space Groups

Structural solutions and refinements of compounds which crystallize in polar space groups have been known for a long time to be problematic. Choosing the wrong polarity can lead to incorrect bond lengths whenever atoms with significant anomalous scattering are present. I have no specific examples where structures with the wrong polarity have been published but the nature of the problem was clearly pointed out some years ago [23]. Besides the polarity issue, these structures are often plagued with elements of pseudosymmetry. This is particularly true whenever the compound contains a single “heavy” atom which dominates the scattering. The basic problem stems from the fact that placing *one* atom, heavy or otherwise, in a general position in a unit cell with polar symmetry often generates additional symmetry elements.

**Fig. 10:**
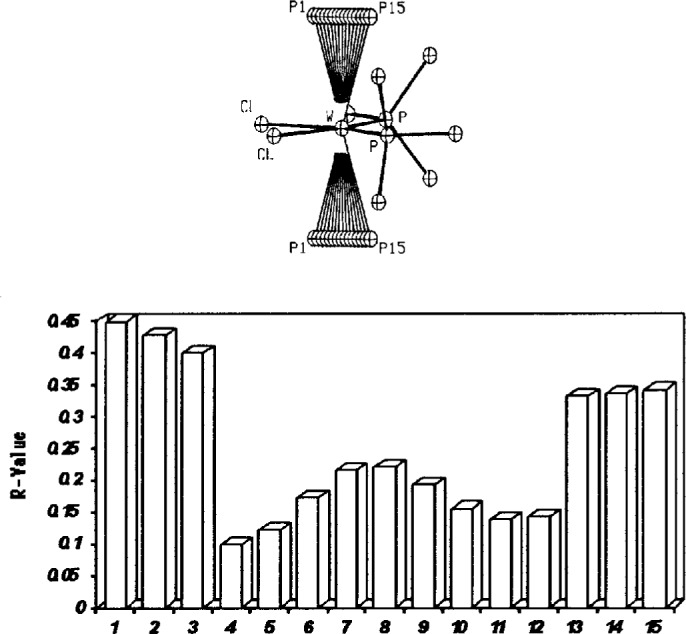
Partial structure of W(PMe_3_)_4_H_2_Cl_2_ (top). The two axial phosphorus atoms (without the methyl groups) were placed at a series of positions from P1 to P15, with the pseudo-mirror being located at P8. The resulting *R* value for each of these positions is shown in the (bottom) graph. In the correct structure, these phosphorus atoms refine to position P4, the real minimum. In the incorrect structure, they refine to position P12, a false minimum. Thus, once an atom is placed at a pseudo-image position, it is trapped there.

Recent examples of this phenomenon include the structures of two tungsten complexes, W(PMe_3_)_4_H_2_Cl_2_ and W(PMe_3_)_4_H_2_F_2_(H_2_O) [24], the former having been corrected before publication and the latter being a reinvestigation of an incorrect structure [25]. The space group in both cases was Cmc2_1_ and wrong structures were readily obtained and refined to reasonable *R* values. The incorrect structures in these cases contained some atoms from the “real” structure and some from the “pseudosymmetric” structure, in this case produced by a pseudo mirror plane perpendicular to the *c*-axis. The real structure is produced when the axial phosphine ligands tilt towards the equatorial chloride ligands; the incorrect structure has them tilting toward the equatorial phosphines. When refined, both structures will converge very nicely with the incorrect structure converging to a false minimum. Even if one atom is placed in an incorrect position which differs from the correct position by only an Å or so, the least-squares technique will fail to shift the atom to the correct position. This was clearly demonstrated for the structure of W(PMe_3_)_4_H_2_Cl_2_ where the axial phosphorus atoms (related by a real mirror plane) were manually translated from their incorrect to their correct positions as shown in Fig. 10 [26].

What is particularly worrisome about the incorrect structures in these cases is that neither the thermal parameters nor the bond distances gave any strong indication that the structures were wrong. Close contacts between the hydrogen atoms (if calculated in assumed positions) gave the only warning that something was amiss.

We have found, however, that both the bond distances and the thermal parameters may be quite telling if the data are collected at low-temperature where the high-angle reflections are well represented. We have recently encountered another “problematic” structure involving one heavy atom, Hg, in the same polar space group, Cmc2_1_: [(C_5_H_8_N_2_)_2_Hg]Cl_2_ [27]. Whenever atoms from both the real and pseudosymmetric images were included in the refinement, some of the bond distances and thermal parameters associated with “light” atoms (carbons and nitrogens) of the organic ligands became unreasonable. Also, the hydrogen atoms could not be refined properly when the model contained atoms from each image. When the correct model using all atoms from the same image was refined, not only were the bond distances and thermal parameters reasonable but the refinement of the hydrogen atoms was also possible.

### 3.7 Disorder

**Fig. 11:**
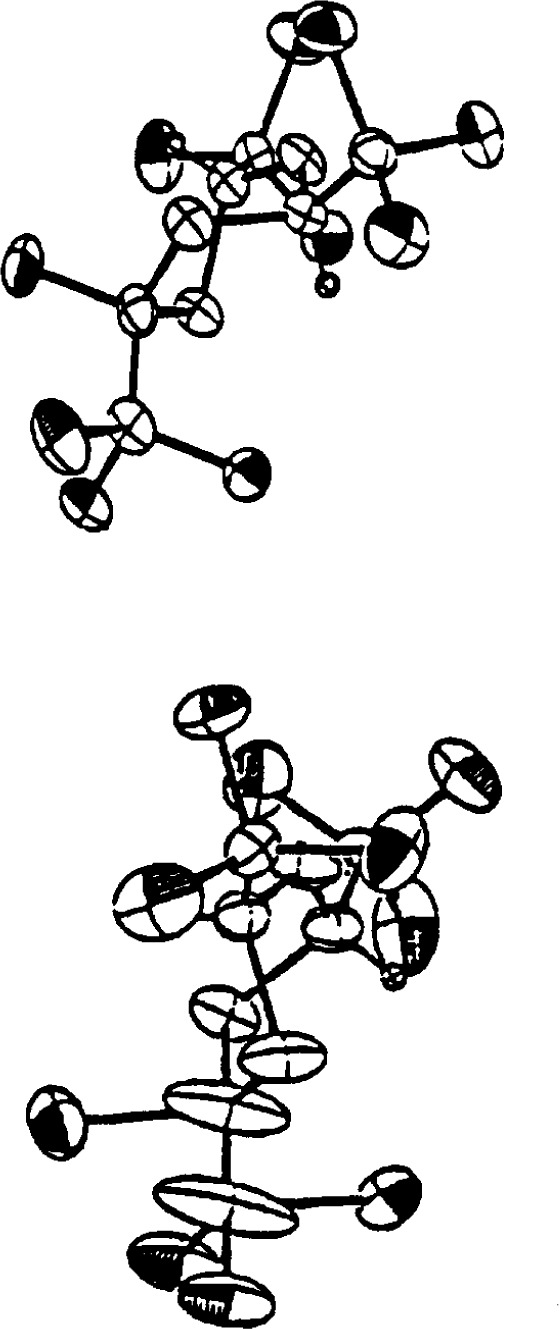
Thermal ellipsoid drawings of the two molecules of *trans*-4-chloro-2,4,6-*tris*(trichloromethyl)-1-*oxa*-3,5-di-thiane. The –CCl-CCl_3_ moieties are located at the bottom left of each drawing. The molecule at the top refined normally; the abnormal molecule on the bottom has severely elongated ellipsoids especially for the two central carbon atoms.

As I have already noted, disorder can have a variety of origins: wrong unit cell, wrong Bravais lattice, partial occupancies, etc. Sometimes, however, a structure can be truly disordered (multiple atomic sites) and the problem be misinterpreted. One such case was the crystal structure of *trans*-4-chloro-2,4,6-*tris*(trichloromethyl)-1-*oxa*-3,5-dithiane [28]. This compound was made by adding Cl_2_ across a C=C of a precursor molecule. Its structure has two molecules per asymmetric unit, one of which appeared quite normal: the C=CCl_2_ moiety had been converted to Cl-C-C-Cl_3_ with typical C–Cl distances of 1.79 Å and a C–C bond length of 1.55 Å The second molecule, however, appeared to have been caught mid-stream in its reaction with the Cl_2_ to give a Cl–C=CCl_2_–Cl moiety where the C–Cl bonds were now greater than 2.07 Å and the central carbon-carbon bond was 1.28 Å. These authors either didn’t look at the thermal ellipsoids or ignored them. Shown in Fig. 11 are the ORTEP drawings of the molecules given as part of a reinvestigation of this structure [29].

**Fig. 12:**
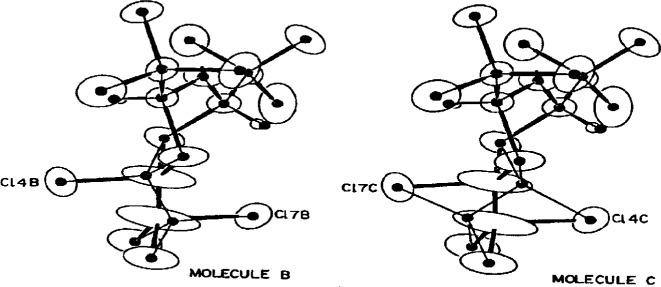
The disordered model, represented by the dots (atom positions) and thin lines (bonds), used to refine the second molecule.

The large ellipsoids of the carbon atoms in the second molecule are clearly indicative of a disorder. When modeled correctly, as shown in Fig. 12, all the bond distances are close to their expected values.

### 3.8 Decomposition

**Fig. 13:**
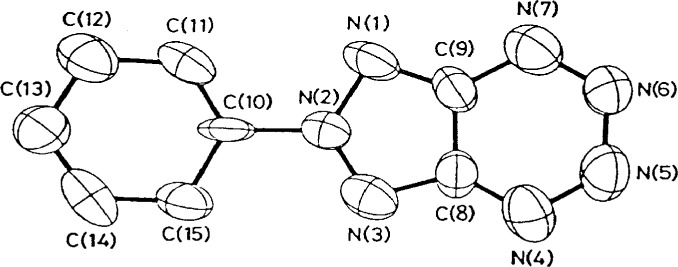
Thermal ellipsoid drawing of 6-phenyl[1,2,3]triazolo[4,5-e]-1,2,3,4-tetrazine.

I have always had the impression that crystallographers generally consider decomposition to be a rather benign source of intensity errors, errors that are easily corrected by using a set of intensity standards that are measured periodically during the data collection process. When a crystal decomposes, it is the high-angle reflections, and hence the “resolution” of the structure which suffer most. Invariably, the standards used to correct for the loss of intensity are low-angle reflections. Thus, the correction is generally inappropriate. Even if reflections from all angles are selected as standards, the assumption that all reflections within a small range of two-theta are affected equally by the decomposition is only roughly valid. An example of the problems that can arise is shown in Fig. 13, a structure which as originally reported, refined to *R* = 0.168 after correcting for a 15.1 % loss in intensity [30]. Chemically-equivalent bond lengths are quite disparate: N(1)–N(2), 1.359 Å; N(2)–N(3), 1.299 Å: N(7)–C(9), 1.348 Å; N(4)–C(8), 1.268 Å.

These same authors later collected data with the crystal cooled to 153 K [31]. At this temperature, there was no decomposition and the structure refined reasonably well (*R* = 0.069): all of the chemically equivalent bond distances differed by less than 3 sigma.

### 3.9 *Fuzzy* Structures

**Fig. 14:**
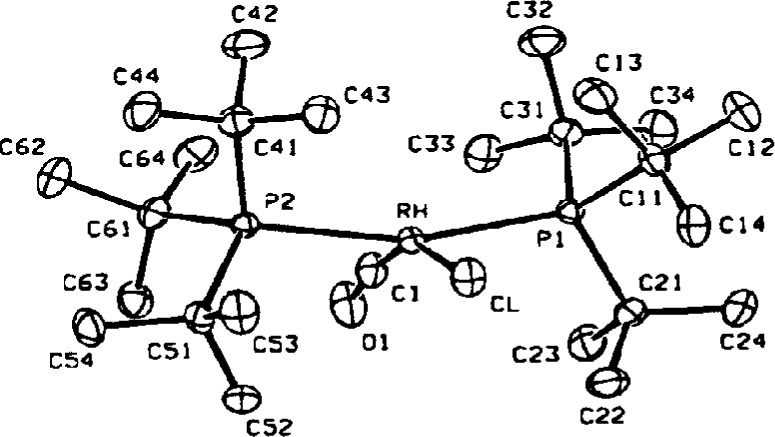
Distorted structure of [P(*t*-Bu)_3_]_2_Rh(CO)Cl.

The problem with *fuzzy* structures is that it is difficult to decide whether they are interesting structures or wrong structures. I would like to present two examples to illustrate the point. The first is the structure of [P(*t*-Bu)_3_]_2_Rh(CO)Cl in which the rhodium(I) should have square-planar coordination. As first reported [32], however, the geometry about the Rh atom was that of a flattened tetrahedron. In addition, the Rh–C=O angle was bent, 164.7°, and the C=O distance was ridiculously short: 0.987 Å (Cl contamination?). Since this structure was interesting to me as part of a general study of rhodium compounds, I decided to have a second look at it. The resulting structure, refined with “low-temperature” data, is shown in Fig. 14 [33]. The coordination about the rhodium is, without question, that of a flattened tetrahedron. The Rh–C=O angle is indeed bent, 167.3°, although the C=O distance is now far more reasonable at 1.150 Å. Because the positions of the hydrogen atoms could be refined, it is now clear that the the severe distortion in this complex is simply the result of steric interactions between the bulky *tris*(*t*-butyl)phosphine ligands and the CO and Cl ligands. Where unusual structures are concerned, only a *quality* structure gives one the level of confidence needed to proclaim that it indeed has unique features.

It is very common to rush through the data collection process either by using high scan speeds or by limiting the data collection to the low-angle reflections. Both of these limit the resolution of the structure since the weaker high-angle reflections are then not included in the refinement. Even those who advocate using all of the data in their refinements are deluding themselves if the high-angle intensities all have near zero values. One example of a low-resolution structure is that of 3,4-dimethoxy-cinnamic acid which was originally solved and refined using only 725 reflections [34]. The structure is triclinic, $P\bar{1}$, with a *Z* value of 4. With two molecules per asymmetric unit, a refinement using anisotropic thermal parameters would have produced a data/parameter ratio of something less than 3. In addition, a close inspection of the structure showed that the two crystallographically independent molecules were related by a pseudo-center of symmetry. As a result, the refinement was unstable, characterized by high correlations and nonpositive-definite thermal parameters. Many of the resulting bond lengths were unreasonable.

**Fig. 15:**
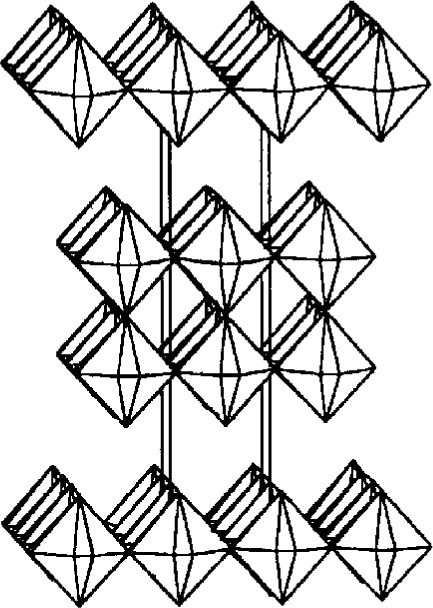
Two molecules of 3,4-dimethoxycinnamic acid which form a hydrogen-bonded pair via a pseudo-center of symmetry. The hydrogen atoms have been drawn with their refined isotropic thermal spheres.

Desiraju brought this structure to my attention because he was interested in resolving whether the refinement was unstable because of the lack of data, the presence of pseudo-centers, or both. Data were collected with the crystal cooled to −100 °C and at room temperature. Refinements using both data sets (2543 and 1893 reflections, respectively, with 367 variables including isotropic hydrogen atoms) converged rapidly with no high correlations between the two molecules [35] Clearly, the original problem with the structure was not the pseudosymmetry, just the lack of data, in particular the high-angle data. Shown in Fig. 15 is the asymmetric unit of this structure refined with data collected at −100 °C.

### 3.10 Structures That Don’t Exist At All

**Fig. 16:**
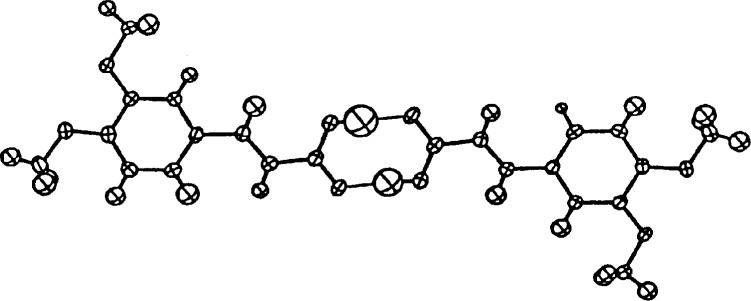
“Ordered” structure of Sr_5_Ir_3_O_11_ showing only alternating single and double layers of IrO_6_ octahedra.

While investigating a series of strontium iridium oxides, one crystal which appeared to have the composition Sr_5_Ir_3_O_11_ was studied [36]. The *c*-axis of this tetragonal cell suggested that it was composed of alternating single and double layers of IrO_6_ octahedra as shown in Fig. 16. Via direct methods and subsequent electron-density maps, the structure solved quite nicely to yield the “expected” model (*R* = 0.050). However, two of the atoms, one iridium and one oxygen, had nonpositive-definite thermal parameters. Subsequently, a careful inspection of the data collection profiles showed that many peaks were not well centered and that the *c*-axis dimension had an unreasonably large esd. Photographic techniques were then employed to show that the crystal was not truly “single”: there were in fact many bizarre reflections, some of which were sharp, some broad, and some of which appeared to be split. These characteristics are commonly found in materials where random stacking of more than one type of layer occurs. Indeed, a high-resolution electron microscopy image showed that the crystal actually consisted of separate regions of single-layered Sr_2_IrO_4_ and double-layered Sr_3_Ir_2_O_7_, randomly interleaved to produce an approximate 50:50 mixture. I have every reason to believe, however, that the “incorrect” structure could easily have been published.

## 4. Conclusions

There are several types of “incorrect” structures that I have not listed among the examples here, although there may be some in my treasure-trove of “improbable structures” culled from the literature over a 25 year period. “Twinned” crystals, for example, that weren’t recognized as such. Cases where serious absorption problems were ignored or improperly corrected for; Diffractometer misalignments; Unrecognized phase transitions during data collections; Misuse of programs like DIFABS; Problems I haven’t even thought of. Anyone interested in learning more about “wrong” structures should not only read Refs. [1, 2, 3, 4 and 29], but should also peruse the titles of crystallographic papers for keywords such as reinvestigation, redetermination and pitfalls. One should also be wary of words such as new, novel and unique when applied to crystal structures; with tens of thousands of structures in the databases (even if I believe that most of them are *fuzzy*), hardly anything is really “new.”

After examining a large number of “wrong” structures, I have come to a number of (sometimes interrelated) conclusions:

1. Wrong structures generally have good *R* values. While the *junk* group is expected to have a large number of incorrect structures, wrong structures are found, more often than I believe is generally recognized, in the *fuzzy* group.
2. Wrong organic and organometallic structures, almost without exception, are based on “room-temperature” intensity data where the high-angle reflections, if measured at all, are largely “unobserved.” These are basically low-resolution structures, particularly with respect to the “light” atoms which have large vibrational amplitudes and which contribute little to the intensity of the high-angle reflections.
3. Wrong structures usually exhibit one or more of three symptoms: unusual bond distances, unreasonable thermal ellipsoids, and/or impossible intra- and interatomic nonbonded contacts.

No *quality* structure has ever been shown to be incorrect. Hydrogen atoms, for example, simply don’t refine well if either the data or the model contain even modest errors. Organic and organometallic structures must generally be refined using “low-temperature” data in order to be placed in this category. The other advantage of cooling crystals is that unreasonable thermal parameters become much more obvious and, thus, wrong structures are much easier to spot and correct. In fact, I am so confident in my concept of a *quality* structure that I would like to challenge readers to find an example of a *quality* structure that is wrong!

There is little excuse for incorrect structures today. Programs exist to check the metric symmetry of unit cells, to average raw data under a variety of symmetry conditions (although absorption effects may still lead to difficulties here) and to look for additional symmetry elements once the structure is complete. The consistency of chemically-equivalent bond distances, particularly among the “lighter” elements, is easily checked. Databases can be used to see if any of bond distances fall out of the expected ranges or if any of the atoms have unknown coordination geometries. Inorganic structures can be analyzed via the bond-valence model [37]. Strange thermal ellipsoids should not be ignored—they signal that something is wrong with the data, the model, or both. By the way, once you become sensitized to strange ellipsoids, you will see them everywhere! Finally, the structure should be checked for close intra- and interatomic distances, particularly among the hydrogen atoms.

## References

[1] Ibers JA, Lide DR, Paul MA (1974). Critical Evaluation of Chemical and Physical Structural Information.

[2] Jones PG (1984). Chem Soc Rev.

[3] Parkin G (1993). Chem Rev.

[4a] (1995). R. E. Marsh has numerous publications related to symmetry corrections, the most recent of which is R. E. Marsh and I. Bernal. Acta Cryst.

[4b] Baur WH, Tillmanns E (1986). Acta Cryst.

[5] Ferraris JP, Andrus RG, Hrncir DC (1989). J Chem Soc, Chem Commun.

[b6-j3harl] 6M. C. Kuchta, H. V. R. Dias, S. G. Bott, and G. Parkin, personal communication on a reevaluation of the structure of [T_p_^t-Bu^]In

[7] Ogle CA, Masterman TC, Hubbard JL (1990). J Chem Soc, Chem Commun.

[8] Dunbar KR, Haefner SC (1992). Inorg Chem.

[9] Harlow RL, Thorn DL, Baker RT, Jones NL (1992). Inorg Chem.

[10] Lingnau R, Straehle J (1988). Angew Chem Int Ed Engl.

[11] Haaland A, Rypdal K, Verne HP, Scherer W, Thiel WR (1994). Angew Chem Int Ed Engl.

[12] Yoon K, Parkin G (1991). J Amer Chem Soc.

[13] Gibson V, McPartlin M (1992). J Chem Soc Dalton Trans.

[14] Miele P, Foulon JD, Hovnanian N, Durand J, Cot L (1992). Eur J Solid State Inorg Chem.

[15] Laxman RK, James AM, Fronczek FR, Maverick AW Inorg Chem.

[16] Randall JJ, Katz L, Ward R (1957). J Amer Chem Soc.

[17] Crawford MK, Subramanian MA, Harlow RL, Fernandez-Baca JA, Wang ZR, Johnston DC (1994). Rhys Rev.

[18] Iball J, Scrimgeour SN (1977). Acta Cryst.

[19] Zanotti G, Del Pra A, Bombieri G, Tamburro AM (1978). Acta Cryst.

[20] Lock CJL, Speranzini RA, Zvagulis M (1980). Acta Cryst.

[21] Schneider JJ, Goddard R, Werner S, Krueger C (1991). Angew Chem Int Ed Engl.

[22] Theopold KH, Kersten JL, Rheingold AL, Casey CP, Wiedenhoefer RA, Hop CECA (1992). Angew Chem Int Ed Engl.

[23] Cruickshank DWJ, McDonald WS (1967). Acta Cryst.

[24] Murphy VJ, Rabinovich D, Parkin G (1995). J Amer Chem Soc.

[25] Minqin C, Prout K (1986). J Struct Chem.

[26] Harlow R (1995). “False Minima Can Cause Crystal Structure Errors,” Science/technology article. S Borman, Chem & Eng News.

[b27-j3harl] 27W. J. Marshall and R. L. Harlow, manuscript in preparation.

[28] Irving A, Irving HMNH (1988). J Crystallogr Spectrosc Res.

[29] Kapon M, Herbstein FH (1995). Acta Cryst.

[30] Kaihoh T, Itoh T, Yamaguchi K, Ohsawa A (1988). J Chem Soc Chem Commun.

[31] Yamaguchi K, Kaihoh T, Itoh T, Ohsawa A (1991). Acta Cryst.

[32] Schumann H, Heisler M, Pickardt J (1977). J Chem Ber.

[33] Harlow RL, Westcott SA, Thorn DL, Baker RT (1992). Inorg Chem.

[34] Desiraju GR, Kamala R, Kumari BH, Sarma JARP (1984). J Chem Soc Perkin Trans.

[35] Desiraju GR, Calabrese JC, Harlow RL (1991). Acta Cryst.

[36] Harlow RL, Li ZG, Marshall WJ, Crawford MK, Subramanian MA (1995). Mat Res Bull.

[37] Brown ID (1992). Acta Cryst.

